# Associations of traditional Chinese medicine body constitution and all-cause mortality in patients with type 2 diabetes mellitus: a prospective cohort study of a Taiwanese medical center

**DOI:** 10.3389/fmed.2023.1320861

**Published:** 2024-01-05

**Authors:** Cheng-Hung Lee, Yi-Chang Su, Shih-Yi Lin, I-Te Lee, Chia-I Tsai, Tsai-Chung Li

**Affiliations:** ^1^Department of Traditional Chinese Medicine, Taichung Veterans General Hospital, Taichung, Taiwan; ^2^Department of Post-Baccalaureate Medicine, College of Medicine, National Chung Hsing University, Taichung, Taiwan; ^3^National Research Institute of Chinese Medicine, Ministry of Health and Welfare, Taipei, Taiwan; ^4^Division of Endocrinology and Metabolism, Department of Internal Medicine, Taichung Veterans General Hospital, Taichung, Taiwan; ^5^Center for Geriatrics and Gerontology, Taichung Veterans General Hospital, Taichung, Taiwan; ^6^School of Medicine, National Yang Ming Chiao Tung University, Taipei, Taiwan; ^7^School of Medicine, Chung Shan Medical University, Taichung, Taiwan; ^8^Department of Public Health, College of Public Health, China Medical University, Taichung, Taiwan; ^9^Department of Healthcare Administration, College of Medical and Health Science, Asia University, Taichung, Taiwan

**Keywords:** traditional Chinese medicine, body constitution, all-cause mortality, type 2 diabetes, glucose control

## Abstract

**Introduction:**

The objective of this study was to investigate associations between baseline body constitutions (BCs) in traditional Chinese Medicine (TCM) and all-cause mortality in Chinese individuals with type 2 diabetes.

**Methods:**

A total of 887 individuals with type 2 diabetes who were enrolled in managed care in 2010 were included. These individuals were followed up until 2015, and their mortality status was determined through the use of Taiwan National Death Datasets. At baseline, BC status of participants, including Yin deficiency, Yang deficiency, and phlegm stasis, was assessed using a well-developed Body Constitutions Questionnaire. Hazard ratios (HR) were calculated using a multivariate Cox proportional hazards model.

**Results:**

During 6807.2 person-years of follow-up of 887 participants, with an average follow-up period of 7.7 years, a total of 190 individuals died, resulting in an incidence density of 0.0279 person-years. Yin deficiency was associated with all-cause mortality (HR, 95% CI: 1.39, 1.02–1.90). This study indicates that individuals diagnosed with Yin deficiency in TCM, characterized by symptoms such as thirst, reduced urine volume, hard stool, and hot flushes, had a 39% higher risk of all-cause mortality.

**Discussion:**

The findings may provide information for TCM practitioners on tailoring treatment plans for persons with type 2 diabetes. No conclusive statements can be made on the basis of the preliminary data presented here. Controlled prospective studies are warranted.

## Introduction

### Background of body constitution in tradition Chinese medicine

Traditional Chinese medicine (TCM) is a popular form of complementary and integrative medicine both in China and worldwide. In Taiwan, it is covered by the National Health Insurance Program. TCM is based on the theory of body constitution (BC), which involves individualized medicine. An individual’s BC is determined by the balance of Yin and Yang within their body. Yang relates to the energy that maintains bodily functions, which can be assessed by regulating interstitial fluid, body temperature, and organ systems’ physiological functions. Yin refers to the delivery of materials to cells through interstitial fluid and blood (1). When external factors or environmental stimuli disrupt the balance, physical symptoms such as loose stool, fatigue, chills, shortness of breath, and increased urine output may indicate Yang deficiency or energy deficiency (2, 3). On the other hand, symptoms such as thirst, reduced urine volume, hard stool, and hot flushes may indicate Yin deficiency or a deficiency in essential materials like body fluids, and blood (2, 3). Additionally, symptoms like numbness in the limbs, dizziness, and chest tightness may indicate phlegm stasis or an imbalance in the dynamic harmony state (4). Studies have shown that individuals with different BC are associated with disease and disease progression (4, 5). TCM practitioners employ varied treatment approaches for patients who share the same disease diagnosis, tailoring their treatments to the individual’s body constitution. This concept, known as “tong bing yi zhi” in Chinese, similar to the concept of individualized medicine, entails prescribing specific herbal treatments based on the patient’s signs and symptoms to achieve the most optimal treatment outcome (5, 6).

### Importance of all-cause mortality as an outcome measure

Infant and young adult mortality rates have decreased over the past two decades due to improvements in public health and medical care (7). In the general population, young adults have a lower mortality risk compared to middle-aged and elderly adults, particularly due to chronic diseases like diabetes and cardiovascular diseases (8). As the population continues to age, it is important to identify the factors that influence mortality in middle-aged and older adults for clinical practice and public health policy. With the rising costs of medical care and healthcare systems driven by demographic changes, preventive services targeting mortality need to be prioritized in order to reduce costs.

### What has not been done in this line of research question

The prevalence and incidence rates of type 2 diabetes have been steadily increasing worldwide, making it a significant public health concern associated with significant clinical and socioeconomic burdens (9). Diabetes is a leading cause of mortality and is linked to both macrovascular and microvascular complications that contribute to premature death. The identification of the factors associated with mortality can be helpful in addressing premature mortality and improving the accuracy of mortality risk estimates. Studies on diabetes care have explored an association between body constitution (BC) and various clinical outcomes, such as health-related quality of life (10), diabetic retinopathy (11), and incident albuminuria (12). However, no previous studies have investigated an association between BC and mortality. Therefore, the objective of this study was to examine associations between baseline BC measured by body constitution questionnaires (BCQs) and risks of all-cause mortality among Chinese individuals with type 2 diabetes.

## Methods

### Study design and participants

The Taichung Diabetic Body Constitution Study (TDBCS) is a hospital-based prospective cohort study that recruited participants with type 2 diabetes aged 18 years and older from the Department of Endocrinology and Metabolism outpatient clinics at Taichung Veterans General Hospital in Taichung, Taiwan. The recruitment period was from February 2010 to February 2011. The patients were selected from the Diabetes Shared Care Network of Taichung Veterans General Hospital. Baseline data collection was conducted during the period from February 2010 to February 2011, and the endpoint for the study was set at September 20, 2015. Type 2 diabetes was diagnosed based on the criteria of the American Diabetes Association, including fasting plasma glucose levels >126 mg/dL, random plasma glucose levels >200 mg/dL with symptoms (such as polydipsia, polyuria, and unexplained weight loss), and 2-h plasma glucose levels >200 mg/dL during an oral glucose tolerance test on two separate occasions. After excluding participants from the original TDBCS who had missing data in the BCQs (with missing items ≥10%), a total of 887 participants diagnosed with type 2 diabetes were included in the study.

### Data collection approach and measurements

All clinical variables and biomarkers of the participants in TDBCS were assessed during a comprehensive health check-up at baseline. These variables included body measurements such as waist circumference and body mass index (BMI), blood pressure measurements including systolic blood pressure (SBP) and diastolic blood pressure (DBP), as well as various blood and urinary tests. Additionally, the participants’ diabetes history, history of comorbidities and complications, and medication use were also recorded. Blood and urinary samples were collected from the participants’ antecubital vein in the morning after a 12-h overnight fast. These samples were then sent for analysis within 4 h. The biomarkers analyzed included HbA1c and fasting plasma glucose (FPG) to assess glucose levels, creatinine to measure kidney function, and lipid profiles including low-density lipoprotein-cholesterol (LDL-C), high-density lipoprotein-cholesterol (HDL-C), and triglycerides (TG). A standardized questionnaire was used to collect self-reported data, which included the BCQ assessing previous or current disease history, medication use, and lifestyle behaviors.

### Measurements for BCQ

The BCQ, developed by Su et al. (3), consists of 44 five-point Likert-type items. The questionnaire measures the physiological state of patients’ BC in terms of Yang deficiency (19 items), Yin deficiency (19 items), and phlegm stasis (16 items). There is some overlap in the items between these three scales. The responses to the five-point questions range from 1 (no occurrence) to 5 (always occurring). The multi-item summative scales for the BCQ, representing Yang deficiency, Yin deficiency, and phlegm stasis, have ranges of 19–95, 19–95, and 16–80, respectively. Higher scores on the BCQ scales indicate a greater deviation from the specific BC being measured. The cut-off points for defining Yang deficiency, Yin deficiency, and phlegm stasis are set at 30.5, 29.5, and 26.5, respectively (2, 13). The BCQ was administered to all participants as a self-report questionnaire by trained interviewers. The interviewers were trained to identify and address any ambiguity, discrepancy, or omission in order to minimize potential error. BCQ has a good factorial validity (2) and internal consistency with Cronbach’s alpha above 0.88 for all three BCQ variables (2).

### Outcome ascertainment

The main outcome variable was all-cause mortality, which was determined from the annual record linkage with the Taiwan National Death Datasets provided by the Taiwan Ministry of Health and Welfare. The linkage was based on basic information such as the personal identification number and date of birth. All patients included in the study were followed up from their index date until August 2021 or until the occurrence of death. The index date refers to the date of entry into the present study.

### Measurements for covariates

The covariates in the study included sociodemographic factors such as age and sex, anthropometric measurements of waist circumference and BMI, blood pressure measurements of SBP and DBP, lifestyle behaviors such as smoking history, alcohol consumption, and physical activity habits, and diabetes-related variables including duration of diabetes and type of anti-hyperglycemia medication. These covariates were obtained through personal interviews and physical check-ups. Biochemical markers, including HbA1c, FPG, creatinine, blood urea nitrogen (BUN), serum glutamic-pyruvic transaminase (SGPT), serum glutamic-oxaloacetic transaminase (SGOT), uric acid, total cholesterol, HDL-C, TG, and LDL-C were analyzed using a biochemical autoanalyzer (Hitachi Labospect 008, LST008, Hitachi High-Technologies Corporation, Tokyo, Japan). The estimated glomerular filtration rate (eGFR) was calculated using the formula suggested by the Chronic Kidney Disease Epidemiology Collaboration (14). The anti-diabetes medication included two main categories, namely, oral medication agents and insulin injection.

### Statistical analysis

Descriptive statistics were used to report the mean and standard deviation for continuous variables. Two-sample *t*-tests were conducted to compare variables between groups. Frequencies and percentages were reported for categorical variables, and the chi-square test was used to assess differences in categorical variables between groups. Bivariate survival analyses were performed using the Kaplan–Meier method to estimate survival curves, and the log-rank test was used to compare the survival functions among BC subgroups (15). Cox proportional hazard models were used to estimate hazard ratios (HRs) and their 95% confidence intervals (CIs). This method allowed for the assessment of an association between the time to an event outcome and a set of explanatory variables (16). Three models were created for the Cox models. Model 1 did not include any covariates. Model 2 included adjustments for sociodemographic characteristics, lifestyle behaviors, BMI, waist, blood pressure, and lipid profile. Model 3 included additional adjustments for diabetes-related factors, such as anti-diabetes medication and diabetic duration, eGFR, albuminuria status, diabetic retinopathy, and cerebrovascular accident. The proportionality assumption was assessed by including an interaction term of BC subgroups and person-time in the Cox models. No statistically significant violation of the assumption was found. Multiple testing was not used in our study because two-group comparisons were made, one outcome variable was considered, and no interim analysis was conducted in the present study. All analyses were conducted using SAS version 9.4 (SAS Institute, Cary, NC). Two-sided *p* values were reported, and statistical significance was considered at *p* < 0.05.

## Results

### Distributions of baseline characteristics

A total of 887 individuals with type 2 diabetes were included in the present study. The average age at baseline was 63.8 (standard deviation: 13.4) years. During the 6807.2 person-years of follow-up period, with a mean (median) follow-up of 7.7 (8.5) years, 190 individuals died, resulting in an incidence density of 0.0279 person-years. The characteristics of the participants according to their survival status are described in Table 1. In decedents, the mean values of age, waist circumference, HbA1c, diabetes duration, SBP, albumin-to-creatinine ratio, serum creatinine, and eGFR were higher compared to those in patients who were alive. Additionally, the mean value of HDL-C was lower in decedents than in patients who were alive. Male individuals, those with BCs of Yin deficiency and phlegm stasis, no exercise habits, insulin use, albuminuria, diabetic retinopathy, and cerebrovascular accident were found to have a lower probability of survival compared to individuals who did not have these characteristics.

**Table 1:** Sociodemographic characteristics, body constitution, lifestyle behaviors, diabetes-related factors, biomarkers, and comorbidities according to survival status.

	Decedents	Survivors	
Variables	(*n* = 190)	(*n* = 697)	*p* value
**Sociodemographic factors**			
Age (years)	73.6 (10.7)	61.1 (12.8)	<0.001
**Gender**			
Female	57 (15.0%)	324 (85.0%)	<0.001
Male	133 (26.3%)	373 (73.7%)	
BMI (kg/m^2^)	25.5 (3.5)	25.7 (4.0)	0.42
Waist (cm)	91.0 (10.3)	88.8 (10.9)	0.01
**Body constitution**			
**Yang deficiency**			
No	164 (21.0%)	618 (79.0%)	0.37
Yes	26 (24.8%)	79 (75.2%)	
**Yin deficiency**			
No	120 (18.5%)	527 (81.5%)	<0.001
Yes	70 (29.2%)	170 (70.8%)	
**Phlegm stasis**			
No	154 (20.1%)	614 (79.9%)	0.01
Yes	36 (30.3%)	83 (69.7%)	
**Lifestyle behaviors**			
**Smoke history**			
No	184 (21.8%)	659 (78.2%)	0.20
Yes	6 (13.6%)	38 (86.4%)	
**Alcohol history**			
No	187 (21.8%)	672 (78.2%)	0.16
Yes	3 (10.7%)	25 (89.3%)	
**Exercise habits**			
No	52 (27.5%)	137 (72.5%)	0.02
Yes	138 (19.8%)	559 (80.2%)	
**Diabetes-related factors**			
FPG (mg/dL)	150.6 (52.9)	143.2 (46.2)	0.08
HbA1c (%)	7.9 (1.7)	7.6 (1.5)	0.03
Diabetes duration (years)	11.4 (9.3)	8.6 (7.7)	<0.001
**OHA**			
No	9 (22.0%)	32 (78.0%)	0.93
Yes	181 (21.4%)	665 (78.6%)	
**Insulin usage**			
No	130 (19.3%)	544 (80.7%)	0.006
Yes	60 (28.2%)	153 (71.8%)	
**Lipid profile**			
TC (mg/dL)	172.6 (40.5)	174.1 (34.5)	0.63
TG (mg/dL)	144.7 (108.5)	148.0 (125.2)	0.723
HDL (mg/dL)	50.5 (13.3)	52.9 (15.0)	0.05
LDL (mg/dL)	105.9 (37.1)	106.0 (30.3)	0.98
**Blood pressure**			
SBP (mmHg)	133.5 (15.3)	131.1 (14.3)	0.04
DBP (mmHg)	77.0 (10.9)	77.8 (8.7)	0.35
**Renal parameters**			
Albumin-to-creatinine ratio (mg/g)	46.0 (91.4)	15.2 (58.4)	<0.001
Creatinine (mg/dL)	1.5 (0.8)	1.1 (0.5)	<0.001
eGFR (mL/min/1.73m^2^)	54.5 (20.4)	71.4 (21.4)	<0.001
**Albuminuria**			
No (<30 mg/g)	58 (12.2%)	419 (87.8%)	<0.001
Yes (≥30 mg/g)	120 (32.1%)	254 (67.9%)	
**Diabetic retinopathy**			
No	94 (17.7%)	438 (82.3%)	<0.001
Yes	96 (27.0%)	259 (73.0%)	
**Cerebrovascular accident**			
No	174 (20.4%)	680 (79.6%)	0.001
Yes	16 (48.5%)	17 (51.5%)	

Data were presented in terms of the mean (SD) for continuous variables or number (%) for categorical variables. *p* values were calculated using Chi-square test for categorical variables and *t*-test for continuous variables.

BMI, body mass index; FPG, fasting plasma glucose; OHA, oral hypoglycemic agent; TC, total cholesterol; TG, triglycerides; LDL, low-density lipoprotein; HDL, high-density lipoprotein; SBP, systolic blood pressure; DBP, diastolic blood pressure; eGFR, estimated glomerular filtration rate.

### Associations of BCs with all-cause mortality

**Figure 1:**
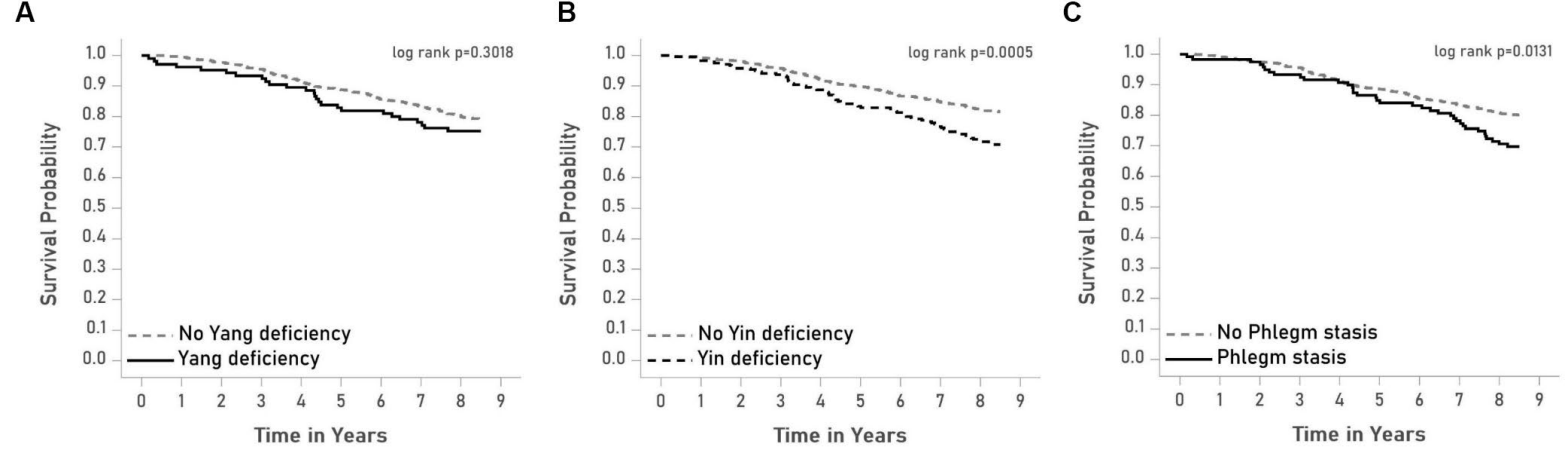
Survival curves of all-cause mortality according to the subgroups of **(A)** Yang deficiency, **(B)** Yin deficiency, and **(C)** Phlegm stasis.

The Kaplan–Meier method was used to estimate survival probability for all-cause mortality within subgroups of BCs of Yang deficiency, Yin deficiency, and phlegm stasis, as shown in Figure 1. The HRs estimated from the three models of all-cause mortality according to Yang deficiency subgroups are presented in Table 2. Yang deficiency was not significant in the model without adjustment, and this remained non-significant even after considering sociodemographic characteristics, lifestyle behaviors, waist circumference, blood pressure, and lipid profiles (model 2), as well as diabetes-related factors, eGFR, albuminuria, diabetic retinopathy, and cerebrovascular accident (model 3). Yin deficiency showed a significant association with all-cause mortality in the model without adjustment (HR, 95% CI: 1.68, 1.25–2.26). This association remained significant after further considering factors related to all-cause mortality, although its effect was attenuated when lifestyle behaviors and diabetes-related factors were included in the multivariate model (HR, 95% CI: 1.39, 1.02–1.90, Table 3). Phlegm stasis was significantly associated with all-cause mortality in the model without adjustment (HR, 95% CI: 1.58, 1.10–2.27), and this association remained significant after further considering sociodemographic factors, lifestyle behaviors, and blood pressure and lipid profiles (HR, 95% CI: 1.39, 1.02–1.90). However, its effect became borderline after multivariate adjustment (HR, 95% CI: 1.43, 0.95–2.15, Table 4). Other significant factors associated with all-cause mortality were age (HR, 95% CI: 1.08, 1.06–1.10), gender (HR, 95% CI: 1.95, 1.35–2.81), exercise habits (HR, 95% CI: 0.65, 0.46–0.93), HbA1c (HR, 95% CI: 1.14, 1.01–1.29), eGFR (HR, 95% CI: 0.99, 0.98–0.99), and albuminuria (HR, 95% CI:1.58, 1.12–2.24). We conducted a sensitivity analysis by excluding individuals with stroke (*n* = 33) and end-stage renal disease (*n* = 10) in order to improve the homogeneity of the study subjects and test the robustness of our findings. The results of the sensitivity analysis showed that the findings of the study remained similar to those of the original analysis, indicating the robustness of our results. The HRs for Yang deficiency and phlegm stasis were not significantly associated with all-cause mortality, while Yin deficiency remained significantly associated with all-cause mortality (HR, 95% CI: 1.48 [1.07–2.06], *p* < 0.05).

**Table 2:** Cox’s proportional hazards model: Yang deficiency.

	HR (95%CI)		
Variables	Model 1	Model 2	Model 3
**Body constitution**			
Yang deficiency	1.24 (0.82–1.88)	1.44(0.93–2.20)	1.31 (0.83–2.07)
**Sociodemographic factors**			
Age (years)	1.09 (1.07–1.10)***	1.09 (1.07–1.11)***	1.08 (1.06–1.10)***
Male	1.88 (1.38–2.56)***	1.71 (1.19–2.45)**	1.95 (1.34–2.83)***
**Lifestyle behaviors**			
Smoke history	0.60 (0.27–1.35)	1.15 (0.49–2.68)	0.87 (0.34–2.22)
Alcohol history	0.46 (0.15–1.45)	0.41 (0.12–1.37)	0.66 (0.20–2.23)
Exercise habits	0.67 (0.49–0.92)*	0.54 (0.38–0.75)***	0.64 (0.45–0.90)*
BMI (kg/m^2^)	0.99 (0.95–1.02)	1.00 (0.94–1.07)	1.00 (0.93–1.07)
Waist (cm)	1.02 (1.00–1.03)*	1.00 (0.98–1.03)	1.00 (0.98–1.02)
**Blood pressure**			
SBP (mmHg)	1.01 (1.00–1.02)*	1.00 (0.99–1.02)	1.01 (0.99–1.02)
DBP (mmHg)	0.99 (0.98–1.01)	1.01 (0.99–1.03)	1.00 (0.98–1.03)
**Lipid profile**			
TC (mg/dL)	1.00 (0.99–1.00)	1.01 (1.00–1.02)	1.01 (1.00–1.02)
TG (mg/dL)	1.00 (1.00–1.00)	1.00 (1.00–1.00)	1.00 (1.00–1.00)
HDL (mg/dL)	0.99 (0.98–1.00)	1.00 (0.98–1.01)	1.00 (0.98–1.01)
LDL (mg/dL)	1.00 (1.00–1.00)	0.99 (0.98–1.00)	0.99 (0.98–1.00)
**Diabetic factors**			
FPG (mg/dL)	1.00 (1.00–1.01)		1.00 (1.00–1.00)
HbA1c (%)	1.10 (1.01–1.19)*		1.15 (1.01–1.30)*
Diabetes duration (years)	1.04 (1.02–1.05)***		1.00 (0.98–1.02)
OHA	0.94 (0.48–1.83)		0.79 (0.34–1.83)
Insulin usage	1.58 (1.17–2.15)**		1.49 (1.03–2.17)*
**Renal parameters**			
eGFR (mL/min/1.73m^2^)	0.97 (0.96–0.97)***		0.99 (0.98–0.99)**
Albuminuria ≥ 30	2.97 (2.17–4.07)***		1.60 (1.13–2.27)**
Diabetic retinopathy	1.68 (1.27–2.24)***		1.13 (0.83–1.55)
Cerebrovascular accident	2.71 (1.62–4.53)***		1.29 (0.73–2.29)

Model 1 is unadjusted.

Model 2 is adjusted for sociodemographic characteristics, lifestyle behaviors, BMI, waist, blood pressure and lipid profile.

Model 3 is further adjusted for diabetic factors, renal parameters, and diabetic retinopathy and cerebrovascular accident.

BMI, body mass index; SBP, systolic blood pressure; DBP, diastolic blood pressure; TC, total cholesterol; TG, triglycerides; LDL, low-density lipoprotein; HDL, high-density lipoprotein; FPG, fasting plasma glucose; OHA, oral hypoglycemic agent; eGFR, estimated glomerular filtration rate.

*p < 0.05; **p < 0.01; ***p < 0.001.

**Table 3:** Cox’s proportional hazards model: Yin deficiency.

	HR (95%CI)		
Variables	Model 1	Model 2	Model 3
**Body constitution**			
Yin deficiency	1.68 (1.25–2.26)***	1.50 (1.11–2.03)**	1.39 (1.02–1.90)*
**Sociodemographic factors**			
Age (years)	1.09 (1.07–1.10)***	1.09 (1.07–1.10)***	1.08 (1.06–1.10)***
Male	1.88 (1.38–2.56)***	1.69 (1.18–2.40)**	1.92 (1.33–2.77)***
**Lifestyle behaviors**			
Smoke history	0.60 (0.27–1.35)	1.16 (0.50–2.69)	0.90 (0.35–2.30)
Alcohol history	0.46 (0.15–1.45)	0.41 (0.12–1.36)	0.66 (0.20–2.24)
Exercise habits	0.67 (0.49–0.92)*	0.54 (0.38–0.75)***	0.64 (0.45–0.92)*
BMI (kg/m^2^)	0.99 (0.95–1.02)	1.00 (0.93–1.07)	0.99 (0.93–1.07)
Waist (cm)	1.02 (1.00–1.03)*	1.00 (0.98–1.03)	1.00 (0.98–1.02)
**Blood pressure**			
SBP (mmHg)	1.01 (1.00–1.02)*	1.00 (0.99–1.02)	1.01 (1.00–1.02)
DBP (mmHg)	0.99 (0.98–1.01)	1.01 (0.99–1.03)	1.01 (0.98–1.03)
**Lipid profile**			
TC (mg/dL)	1.00 (0.99–1.00)	1.01 (1.00–1.02)	1.01 (1.00–1.02)
TG (mg/dL)	1.00 (1.00–1.00)	1.00 (1.00–1.00)	1.00 (1.00–1.00)
HDL (mg/dL)	0.99 (0.98–1.00)	1.00 (0.98–1.01)	1.00 (0.98–1.01)
LDL (mg/dL)	1.00 (1.00–1.00)	0.99 (0.98–1.00)	0.99 (0.98–1.00)
**Diabetic factors**			
FPG (mg/dL)	1.00 (1.00–1.01)		1.00 (1.00–1.00)
HbA1c (%)	1.10 (1.01–1.19)*		1.14 (1.01–1.29)*
Diabetes duration (years)	1.04 (1.02–1.05)***		1.00 (0.99–1.02)
OHA	0.94 (0.48–1.83)		0.74 (0.32–1.70)
Insulin usage	1.58 (1.17–2.15)**		1.46 (1.01–2.11)*
**Renal parameters**			
eGFR (mL/min/1.73m^2^)	0.97 (0.96–0.97)***		0.99 (0.98–0.99)**
Albuminuria ≥ 30	2.97 (2.17–4.07)***		1.63 (1.16–2.30)**
Diabetic retinopathy	1.68 (1.27–2.24)***		1.09 (0.80–1.49)
Cerebrovascular accident	2.71 (1.62–4.53)***		1.27 (0.72–2.27)

Model 1 is unadjusted.

Model 2 is adjusted for sociodemographic characteristics, lifestyle behaviors, BMI, waist, blood pressure and lipid profile.

Model 3 is further adjusted for diabetic factors, renal parameters, and diabetic retinopathy and cerebrovascular accident.

BMI, body mass index; SBP, systolic blood pressure; DBP, diastolic blood pressure; TC, total cholesterol; TG, triglycerides; LDL, low-density lipoprotein; HDL, high-density lipoprotein; FPG, fasting plasma glucose; OHA, oral hypoglycemic agent; eGFR, estimated glomerular filtration rate.

*p < 0.05; **p < 0.01; ***p < 0.001.

**Table 4:** Cox’s proportional hazards model: Phlegm stasis.

	HR (95%CI)		
Variables	Model 1	Model 2	Model 3
**Body constitution**			
Phlegm deficiency	1.58 (1.10–2.27)*	1.59 (1.07–2.37)*	1.43 (0.95–2.16)
**Sociodemographic factors**			
Age (years)	1.09 (1.07–1.10)***	1.09 (1.07–1.11)***	1.08 (1.06–1.10)***
Male	1.88 (1.38–2.56)***	1.73 (1.21–2.48)**	1.95 (1.35–2.81)***
**Lifestyle behaviors**			
Smoke history	0.60 (0.27–1.35)	1.12 (0.48–2.62)	0.87 (0.34–2.21)
Alcohol history	0.46 (0.15–1.45)	0.41 (0.12–1.37)	0.68 (0.20–2.28)
Exercise habits	0.67 (0.49–0.92)*	0.55 (0.39–0.78)***	0.65 (0.46–0.93)*
BMI (kg/m^2^)	0.99 (0.95–1.02)	1.01 (0.94–1.08)	1.00 (0.93–1.07)
Waist (cm)	1.02 (1.00–1.03)*	1.00 (0.98–1.02)	0.99 (0.97–1.02)
**Blood pressure**			
SBP (mmHg)	1.01 (1.00–1.02)*	1.00 (0.99–1.02)	1.01 (0.99–1.02)
DBP (mmHg)	0.99 (0.98–1.01)	1.01 (0.99–1.03)	1.01 (0.99–1.03)
**Lipid profile**			
TC (mg/dL)	1.00 (0.99–1.00)	1.01 (1.00–1.02)	1.01 (1.00–1.02)
TG (mg/dL)	1.00 (1.00–1.00)	1.00 (1.00–1.00)	1.00 (1.00–1.00)
HDL (mg/dL)	0.99 (0.98–1.00)	1.00 (0.98–1.01)	1.00 (0.98–1.01)
LDL (mg/dL)	1.00 (1.00–1.00)	0.99 (0.98–1.00)	0.99 (0.98–1.00)
**Diabetic factors**			
FPG (mg/dL)	1.00 (1.00–1.01)		1.00 (1.00–1.00)
HbA1c (%)	1.10 (1.01–1.19)*		1.14 (1.01–1.29)*
Diabetes duration (years)	1.04 (1.02–1.05)***		1.00 (0.99–1.02)
OHA	0.94 (0.48–1.83)		0.77 (0.34–1.78)
Insulin usage	1.58 (1.17–2.15)**		1.44 (0.99–2.09)
**Renal parameters**			
eGFR (mL/min/1.73m^2^)	0.97 (0.96–0.97)***		0.99 (0.98–0.99)**
Albuminuria ≥ 30	2.97 (2.17–4.07)***		1.58 (1.12–2.24)**
Diabetic retinopathy	1.68 (1.27–2.24)***		1.14 (0.83–1.55)
Cerebrovascular accident	2.71 (1.62–4.53)***		1.35 (0.76–2.41)

Model 1 is unadjusted.

Model 2 is adjusted for sociodemographic characteristics, lifestyle behaviors, BMI, waist, blood pressure and lipid profile.

Model 3 is further adjusted for diabetic factors, renal parameters, and diabetic retinopathy and cerebrovascular accident.

BMI, body mass index; SBP, systolic blood pressure; DBP, diastolic blood pressure; TC, total cholesterol; TG, triglycerides; LDL, low-density lipoprotein; HDL, high-density lipoprotein; FPG, fasting plasma glucose; OHA, oral hypoglycemic agent; eGFR, estimated glomerular filtration rate.

*p < 0.05; **p < 0.01; ***p < 0.001.

### Assessing interactions of covariates on associations between BCs and all-cause mortality

**Figure 2:**
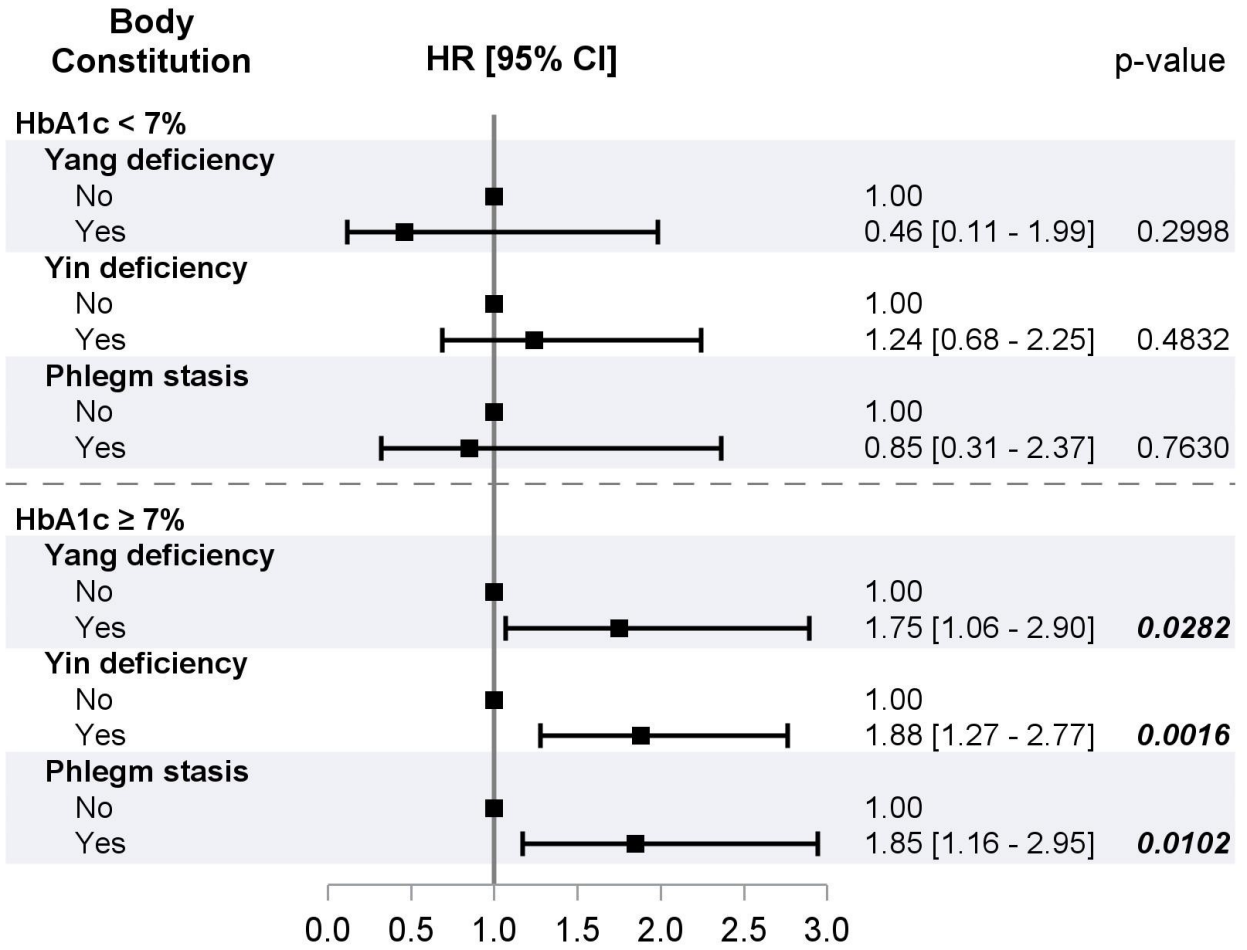
HRs of all-cause mortality for Yang deficiency, Yin deficiency, and phlegm stasis stratified by glucose control status from multivariate Cox proportional hazard models.

The effects of BCs were stratified by gender, age, duration of DM, and glucose control status. No significant interactions of gender, age, and duration of DM with any BCs were observed (all p for interactions > 0.05). Considering the limited sample size, significant associations of Yin deficiency were observed with mortality in persons aged 55 years and older (HR, 95% CI: 1.45, 1.04–2.00) and of phlegm stasis with mortality in persons aged 55 and younger (HR, 95% CI: 0.003, 0.00–0.82). We observed significant interactions of glucose control status with Yang and Yin deficiency (p for interaction = 0.0363 and 0.0379, respectively) and borderline significant interaction of glucose control status with phlegm stasis (p for interaction = 0.0641) (Figure 2). Yang deficiency, Yin deficiency, and phlegm stasis had no significant interactions with mortality in persons with good glucose control, while Yang deficiency, Yin deficiency, and phlegm stasis had a significant positive association with mortality in persons with poor glucose control (HRs, 95% CI: 1.75, 1.06–2.90; 1.88, 1.27–2.77; and 1.85, 1.16–2.95, respectively).

## Discussion

Overall, this study suggests that BCs of Yang deficiency, Yin deficiency, and phlegm stasis are associated with increased mortality in individuals with diabetes, particularly in those with poor glucose control. These BCs may serve as potential prognostic indicators in diabetes management, and further research is needed to understand the underlying mechanisms and develop targeted interventions. The other significant factors associated with mortality were age, gender, physical activity, HbA1c, insulin use, eGFR, and albuminuria. In addition, the study suggests that associations between BCs of Yang deficiency, Yin deficiency, and phlegm stasis with mortality were only significant in individuals with poor glucose control. In persons with poor glucose control, BCs of Yang deficiency, Yin deficiency, or phlegm stasis were associated with 75–88% increase in mortality. On the contrary, in individuals with good glucose control, these BCs were not associated with increased mortality.

In the present study, it was observed that an association between phlegm stasis and all-cause mortality was influenced by diabetes-related variables, renal-related variables such as eGFR and albuminuria, and comorbidities. Among individuals with poor glucose control, there was a significant association between phlegm stasis and all-cause mortality, and this association had a borderline significant interaction with poor glucose control. According to TCM theory, BC is defined as the essential component that makes up a human being and encompasses physiological, psychological, and pathological aspects that characterize an individual’s health. BC is also influenced by both nature and nurture (17). Phlegm stasis arises due to the stagnation of energy flow, leading to the development of watery phlegm or static blood, as explained by TCM theory (1). A previous cross-sectional study has shown that individuals with type 2 diabetes with phlegm stasis BC are more likely to develop albuminuria (11). A prospective cohort study has demonstrated that the interaction between phlegm stasis and glucose control status may affect the development of new-onset albuminuria in individuals with type 2 diabetes. These findings from epidemiologic studies can be explained using TCM theory, as stagnation arises due to difficulties in the transportation of sugar (Yin) by the energy (Yang) among individuals with type 2 diabetes who have poor glucose control. This phenomenon can be understood through the biological mechanisms of vessel obstructions (18) and vascular endothelial dysfunction (19), which hinder the flow of energy. The occurrence of phlegm stasis BC leads to diabetes complications such as renal dysfunction and albuminuria. Therefore, the effect of phlegm stasis is mediated by diabetes-related factors such as FPG and HbA1c, as well as renal-related factors such as eGFR and albuminuria.

The findings of this study have important implications for healthcare providers, as they provide information on potential effects of BCs and their interaction with glucose control status on all-cause mortality. TCM practitioners can consider to tailor treatment plans based on an individual’s body constitution, such as Yang deficiency, Yin deficiency, or phlegm stasis, particularly in patients with poor glucose control. This information can be used to design targeted TCM BC-based education interventions and identify individuals at high risk who could benefit from such interventions to reduce mortality. For example, individuals with Yin deficiency BC may be advised to avoid grilled, fried, and spicy foods, consume less dessert drinks, increase water intake, and include more yin-nourishing foods in their diet, such as pears, yams, water chestnuts, lotus seeds, lilies, fungus, white fungus, wolfberry, and honey.

The strengths of this prospective cohort study include being the first of its kind to examine associations between BCs and all-cause mortality in individuals with type 2 diabetes. Additionally, the study assessed the mediating effect of traditional risk factors, using a hierarchical model approach in TCM research. The findings from the study suggest potential associations between Yin deficiency and all-cause mortality, as well as between phlegm stasis and mortality through diabetes-related factors. Finally, the study utilized a standardized and well-validated instrument to measure BC status, reducing potential measurement error.

It is important to acknowledge the limitations of this study. First, the participants were recruited from a specific population in a medical center in central Taiwan. This may limit the generalizability of the findings to individuals with type 2 diabetes in other clinical settings. The characteristics of the study population, such as higher prevalence of comorbidities and longer diabetes duration, may not be representative of other populations with type 2 diabetes. Therefore, caution should be exercised when applying the results to different populations. Moreover, our prospective cohort study is an observational study, which means that it cannot eliminate the influence of unknown or unmeasured factors due to the absence of randomization. Even though we have made efforts to control for potential confounding variables through covariate adjustment, there may still be residual confounding that could affect the observed associations.

## Conclusion

This study suggests an association between Yin deficiency and all-cause mortality in individuals with type 2 diabetes. These findings can inform TCM practitioners in tailoring treatment plans based on an individual’s body constitution, developing targeted TCM body constitution-based education interventions, and identifying high-risk individuals who could benefit from such interventions to reduce mortality in individuals with type 2 diabetes. No conclusive statements can be made on the basis of the preliminary data presented here. Controlled prospective studies are warranted.

## Data availability statement

The original contributions presented in the study are included in the article/supplementary material, further inquiries can be directed to the corresponding authors.

## Ethics statement

The studies involving humans were approved by Taichung Veterans General Hospital IRB (approved number: C10007). The studies were conducted in accordance with the local legislation and institutional requirements. The participants provided their written informed consent to participate in this study.

## Author contributions

C-HL: Data curation, Formal analysis, Funding acquisition, Methodology, Project administration, Writing – original draft, Writing – review & editing. Y-CS: Methodology, Project administration, Writing – review & editing. S-YL: Data curation, Writing – review & editing. I-TL: Data curation, Writing – review & editing. C-IT: Data curation, Funding acquisition, Investigation, Methodology, Project administration, Writing – original draft, Writing – review & editing. T-CL: Formal analysis, Investigation, Methodology, Writing – original draft, Writing – review & editing.

## Glossary

**Table:** 

TCM	Traditional Chinese medicine
BC	Body constitution
BCQs	Body constitution questionnaires
TDBCS	Taichung diabetic body constitution study
BMI	Body mass index
SBP	Systolic blood pressure
DBP	Diastolic blood pressure
FPG	Fasting plasma glucose
LDL-C	Low-density lipoprotein-cholesterol
HDL-C	High-density lipoprotein-cholesterol
TG	Triglycerides
BUN	Blood urea nitrogen
SGPT	Serum glutamic-pyruvic transaminase
SGOT	Serum glutamic-oxaloacetic transaminase
eGFR	Estimated glomerular filtration rate
HRs	Hazard ratios
CIs	Confidence intervals
